# Presumptive heterophil extracellular traps recognized cytologically in nine reptile patients with inflammatory conditions

**DOI:** 10.1080/01652176.2021.1873453

**Published:** 2021-01-17

**Authors:** A. J. Flanders, R. J. Ossiboff, J. F. X. Wellehan, A. B. Alexander, D. V. E. Fredholm, T. M. Desiderio, N. I. Stacy

**Affiliations:** a Department of Comparative, Diagnostic, and Population Medicine, College of Veterinary Medicine, University of Florida, Gainesville, FL, USA; b Disney’s Animals, Science and Environment, Disney’s Animal Kingdom®, Bay Lake, FL, USA

**Keywords:** Reptile, cytology, extracellular traps, inflammation

## Abstract

**Background:**

Neutrophil extracellular traps (NETs) represent a novel cellular mechanism of antimicrobial defense activity. Intravascular neutrophils produce extracellular web-like structures composed of chromatin, histones, and cytoplasmic granule proteins to attack and kill microbes. They may impact both pathogen and host; NETs correlate strongly with disseminated intravascular coagulation and mortality in critically ill humans. The mechanism was first discovered in human neutrophils in 2004. Presumptive heterophil extracellular traps (HETs) in a non-avian reptile species were first described in blood films of a gopher tortoise with systemic inflammation.

**Objective:**

While prior reports are limited to blood film review and *in vitro* studies, this descriptive case series highlights the cytological identification of presumptive HETs in nine reptile patients.

**Methods:**

Subjects included six gopher tortoises, one blood python *(Python curtus*), one Burmese python (*P. bivittatus),* and one desert king snake (*Lampropeltis getula splendida*). All six gopher tortoises (*Gopherus polyphemus*) had upper respiratory disease with bacterial etiology (including *Helicobacter* sp. and/or *Mycoplasma* sp.), and snakes had upper respiratory tract infection confirmed with serpentovirus (n = 2) or bacterial dermatitis (n = 1).

**Results:**

Cytology samples with identified HETs included tissue imprints (n = 4), nasal discharge (n = 3), an oral swab (n = 1), and a fine needle aspirate of a skin lesion (n = 1). The identification of specific bacterial (n = 6) and/or viral pathogens (n = 2) was notable.

**Clinical relevance:**

To the authors’ knowledge, this is the first report of presumptive HETs recognized in reptile cytology specimens, suggesting an active cellular process *in vivo* in response to systemic inflammation in non-avian reptiles, and contributing to further understanding of extracellular traps in these species.

## 1. Introduction

The formation of neutrophil extracellular traps (NETs) was first described in human neutrophils in 2004 (Brinkmann et al. 2004). Inciting factors include bacteria, fungi, viruses, parasites, cytokines, or compounds *in vitro* such as phorbol myristate acetate (PMA), and hydrogen peroxide (Brinkmann et al. 2004). The neutrophil flattens and adheres to the substrate, most commonly the wall of a blood vessel. Protein kinase C is activated, initiating the NADPH oxidase signaling cascade, producing reactive oxygen species used as substrate for myeloperoxidase (MPO) (Brinkmann and Zychlinsky 2012; Zhao et al. 2015). The release of neutrophil elastase (NE), and the citrullination of histones (arginine changed to citrulline) follow. Decondensed chromatin, histones, and granule proteins (MPO and NE) are released as NETs (Brinkmann and Zychlinsky 2012; Zhao et al. 2015).

NETs have been documented in veterinary patients; however, published studies are limited to date. Experimental NET formation has been associated with antigenic stimulation, including *Escherichia coli* lipopolysaccharides (LPS) with dog neutrophils, *Toxoplasma gondii* with cat neutrophils, and *Streptococcus equi subspecies zooepidemicus*, *Staphylococcus capitis*, and *E. coli* with equine neutrophils (Rebordão et al. 2014; Li et al. 2017; Lacerda et al. 2019). As in humans, NETs have also been observed with non-infectious inflammatory diseases in other animals, including immune-mediated hemolytic anemia in dogs and equine asthma in horses (Vargas et al. 2017; Lawson et al. 2018). Rare reports exist in non-mammalian species. NETs were experimentally induced in fathead minnow (*Pimephales promelas*) neutrophils, and HETs were documented in a West African lungfish with septicemia resulting from *Edwardsiella tarda* (Palic et al. 2007; Rousselet et al. 2018). Chicken heterophils experimentally exposed to hydrogen peroxide and phorbol myristate acetate (PMA) produced whip-like projections, confirmed as HETs via immunocytochemistry and confocal microscopy (Chuammitri et al. 2009). In 2017, presumptive HETs were described in blood films from a free-ranging gopher tortoise (*Gopherus polyphemus*) with systemic inflammation associated with trauma (Stacy et al. 2017).

This report presents a descriptive case series of nine reptile patients with cytological identification of HETs in diagnostic samples and confirmed systemic inflammation.

## 2. Case series

All patients were presented for veterinary evaluation with various clinical signs from 2018 through 2020. These cases were from an examined caseload of 159 reptile cytology samples during this period, including 14 gopher tortoises and 12 snakes. The patients in this case series were of both sexes and presented during various times of the year.

Medical records of each animal were reviewed retrospectively. For some, whole blood chemistries were performed on an Abaxis chemistry analyzer (VETSCAN VS2 Chemistry Analyzer, Abaxis, Union City, CA 94587, USA). Whole-body computed tomography (CT) performed in Cases 3, 4, 5, and 7 was performed using a 160-slice multidetector CT (Toshiba Aquilion Prime, Cannon Medical Systems, Tustin, CA 92780, USA). Cytology samples were stained with Wright’s-Giemsa stain (Harleco®, EMD Millipore, Billerica, Massachusetts 01821, USA). Sets of tissues were fixed in 10% buffered formalin, embedded in paraffin, sectioned at 5 μm, and stained with hematoxylin and eosin per routine methods.

The following provides a summary for each included patient, with pertinent findings of various diagnostic procedures for confirmation of inflammatory conditions.

### 2.1. Case 1

**Figure 1.**
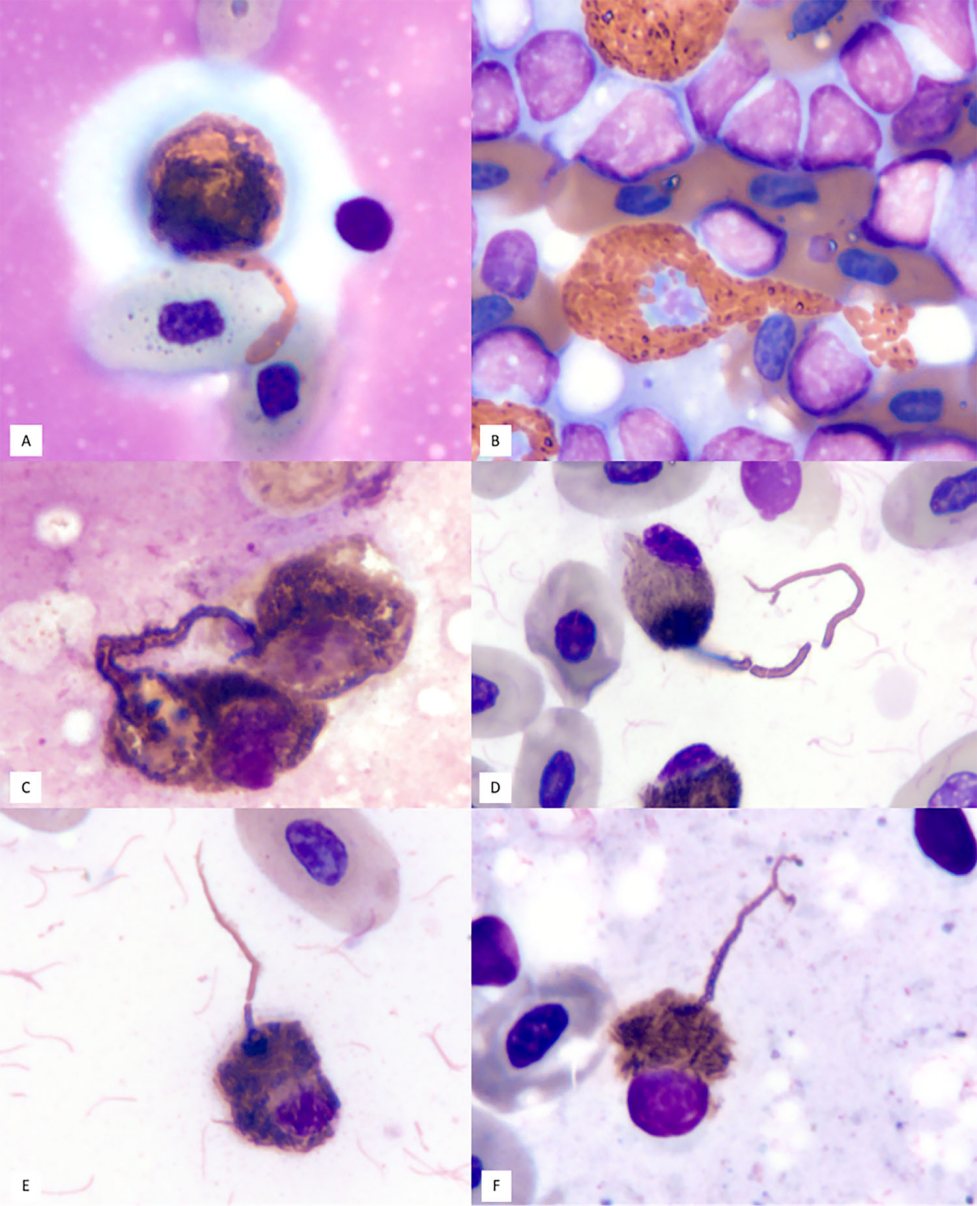
Image composite of heterophils with whip-like projections in cytology samples from reptile patients. A: Blood python (*Python curtus brongersmai*), tissue imprint of spleen (Case 1); B: Blood python (*Python curtus brongersmai*), tissue imprint of spleen (Case 2); C: Gopher tortoise (*Gopherus polyphemus*), oral swab direct smear (Case 3); D, E: Gopher tortoise (*Gopherus polyphemus*), tissue imprint of lung with free cilia from respiratory epithelium in the background; F: Gopher tortoise (*Gopherus polyphemus*), tissue imprint of liver (Case 4). x100 objective, Wright-Giemsa stain.

A captive adult female blood python (*Python curtus brongersmai*) was presented for respiratory distress. Physical examination revealed significant clear, mucoid, oral and nasal secretions, and open-mouth breathing. Hematological abnormalities were absent and plasma biochemistry was not performed. Oral mucosal swabs tested positive for serpentovirus and mycoplasma by polymerase chain reaction (PCR) and sequencing (Hoon-Hanks et al. 2019). Due to the severe clinical condition and poor prognosis, the snake was euthanized. On necropsy, the lungs were grossly dark red, wet, and heavy. A splenic tissue imprint indicated reactive lymphoid hyperplasia with mild histiocytic infiltrate, and few heterophils exhibited whip-like heterophil projections (Figure 1A). Histopathological evaluation identified interstitial, proliferative, and lymphocytic pneumonia with proliferative tracheitis, esophagitis, stomatitis, glossitis, and rhinitis (lymphocytic and granulocytic). The final diagnosis was serpentoviral pneumonia, stomatitis, rhinitis, and tracheitis.

### 2.2. Case 2

A captive adult male Burmese python (*Python bivittatus*) was presented for respiratory distress. Physical examination revealed significant clear, mucoid, oral and nasal secretions and open-mouth breathing. There was marked heterophilia (14 G/L; RI: 0.31-3.99 G/L) based on blood film review, and plasma biochemistry was not performed (Gibbons et al. 2019). Due to the poor clinical condition, the snake was euthanized. On necropsy, the right lung was mottled, dark red, and wet with prominent mucus strands, and the oral cavity was reddened multifocally. A tissue imprint from the spleen revealed reactive lymphoid hyperplasia with a mild mixed inflammatory cell infiltrate and a few heterophils with whip-like heterophil projections (Figure 1B). Histopathology showed interstitial, proliferative, lymphocytic pneumonia, proliferative, segmental tracheitis, hepatic pigmented macrophage center hypertrophy and hyperplasia, proliferative stomatitis, and proliferative, lymphocytic rhinitis. Similar to Case 1, the final diagnosis was serpentoviral pneumonia, stomatitis, rhinitis, and tracheitis.

### 2.3. Case 3

An adult male free-ranging gopher tortoise (*Gopherus polyphemus*) was presented for not moving for an extended period of time. On initial examination, the tortoise had dull mentation with its eyes closed, increased respiratory effort, and audible wheeze. Blood analyses was not performed. A whole-body CT scan revealed bilaterally narrowed nasal turbinates and atrophy of lung tissue. Due to deteriorating clinical condition, euthanasia was elected. On necropsy, the left naris was markedly stenotic with an associated scant amount of clear, watery to mucoid fluid. There was a small amount of cloudy, mucoid material in the oral cavity, and the mucous membranes were tacky. The lungs were diffusely red and heavy with red, clear, watery fluid oozing from the pulmonary parenchyma. Oral swab cytology revealed moderate lymphoplasmacytic and heterophilic inflammation with few heterophil projections and a low number of extracellular mixed bacilli (Figure 1C). On histopathology, there was lymphocytic and heterophilic pneumonia with mucosa-associated lymphoid tissue (MALT) hyperplasia, erosive and heterophilic tracheitis, heterophilic and lymphocytic ulcerative conjunctivitis, necrotizing, heterophilic, blepharitis, ulcerative, heterophilic and lymphoplasmacytic rhinitis with edema intralesional bacteria, and stenosis. Gram stain and Fite’s Acid Fast stain of the right naris revealed a moderate number of Gram negative, non-acid fast rods (top differential being *Mycoplasma* spp.) infection. This animal was *Helicobacter* sp. negative by qPCR and *Mycoplasma* sp. positive by qPCR (Desiderio et al. 2020). The final diagnosis was ulcerative, heterophilic and lymphocytic rhinitis with bilateral stenotic nares, bilateral necro-ulcerative blepharoconjunctivitis, interstitial pneumonia, and thin body condition.

### 2.4. Case 4

**Figure 2.**
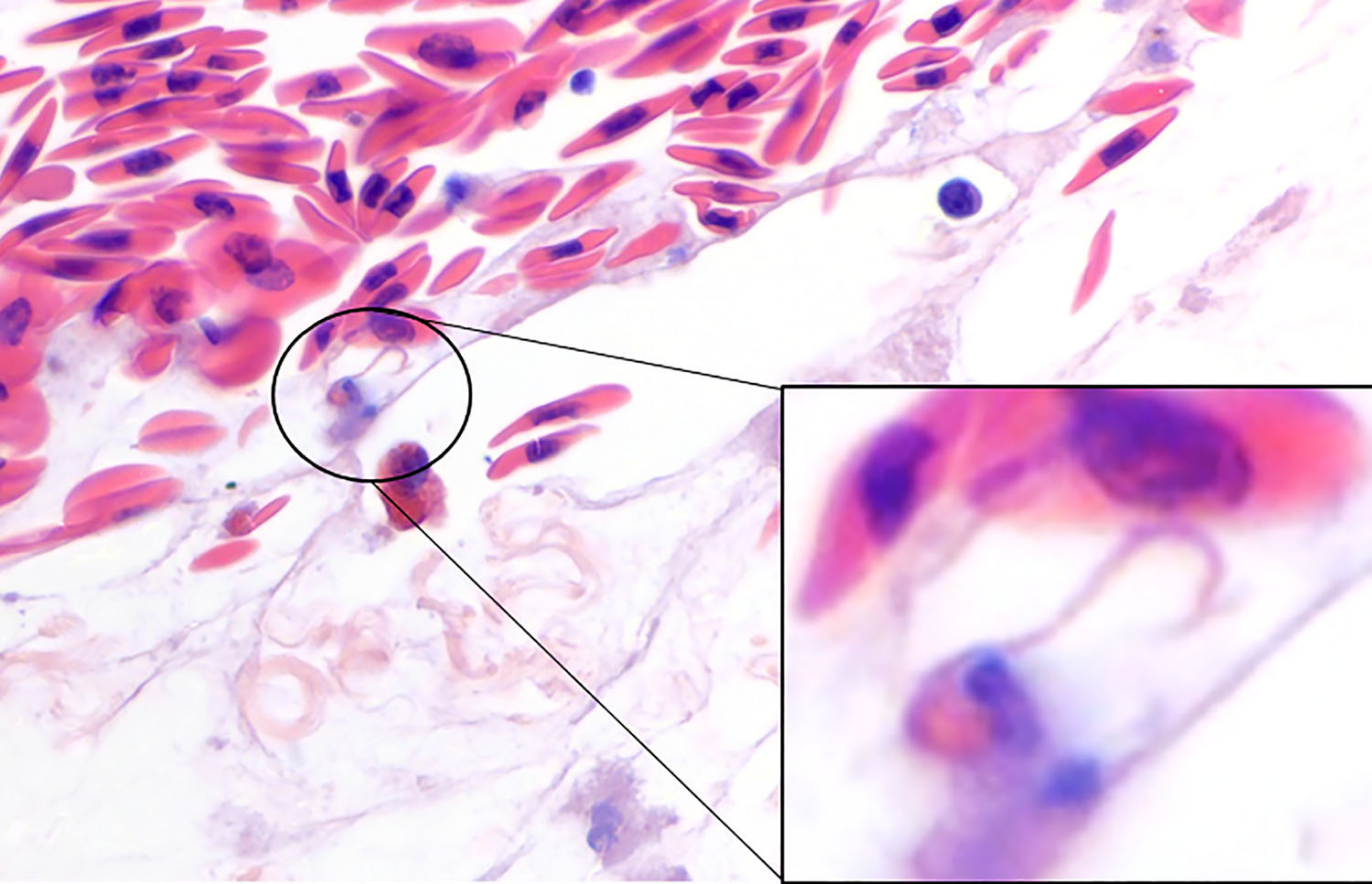
Tissue section of lung of gopher tortoise (*Gopherus polyphemus*) with a single heterophil projection observed histologically. x60 objective, Hematoxylin & Eosin stain.

An adult female gopher tortoise (*G. polyphemus*) from a zoological facility was presented after staff observed increased respiratory noises, effort, and nasal discharge. There were no notable hematology or plasma biochemical abnormalities. Whole-body CT scan revealed mild septal thickening throughout the lung fields. Cytology of nasal discharge identified mild heterophilic, histiocytic, and lympho-plasmacytic rhinitis and rare spirilliform bacteria (presumptive *Helicobacter* sp.). Due to the patient’s poor clinical condition, euthanasia was elected. On necropsy, there was a large amount of white froth in the lower airways; the lungs were wet and oozed a clear, serous fluid. Cytology of lung tissue imprints identified heterophilic, histiocytic, lymphoplasmacytic, and eosinophilic inflammation with occasional heterophil projections (Figure 1D,E). Similarly, cytology of the liver parenchyma revealed reactive lymphoid hyperplasia with frequent mast cells, extramedullary hematopoiesis, and occasional heterophil projections (Figure 1F). Histopathology showed lymphocytic and granulocytic interstitial pneumonia with a single histologically observed heterophil projection (Figure 2), tracheitis, and rhinitis with lymphoplasmacytic and histiocytic bronchitis, lymphoplasmacytic esophagitis, granulomatous gastritis, and oropharyngeal lymphoid hyperplasia. Choanal swab from this animal was positive for a *Helicobacter* sp. by qPCR (Desiderio et al. 2020). The final diagnosis was rhinitis, tracheitis, bronchitis, and pneumonia (lymphocytic and granulocytic) with pulmonary edema.

### 2.5. Case 5

**Figure 3.**
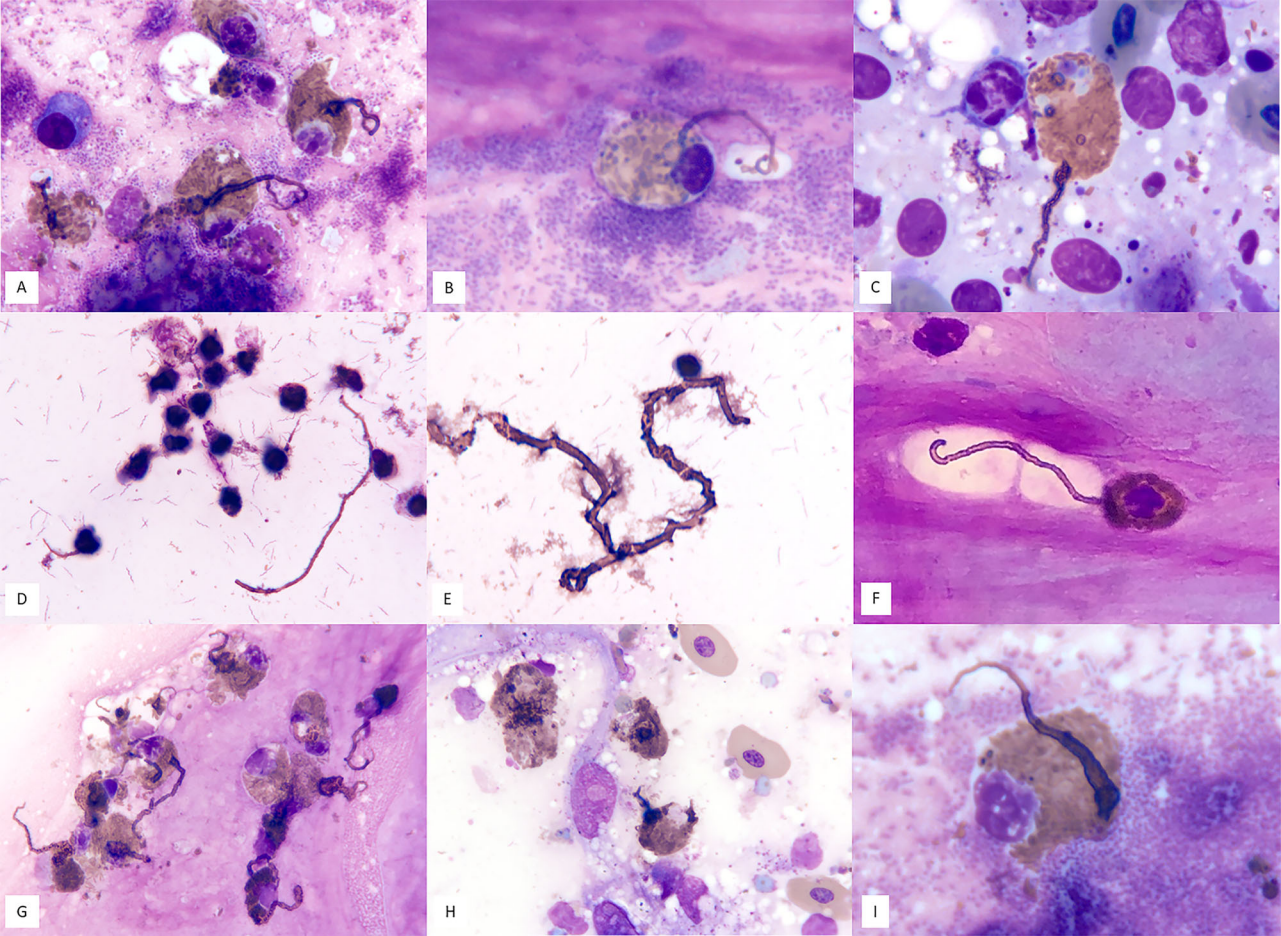
Image composite of heterophils with whip-like projections in cytology samples from reptile patients. A, B: Gopher tortoise (*Gopherus polyphemus*), tissue imprint of oropharynx (Case 5); C: Gopher tortoise (*Gopherus polyphemus*), tissue imprint of spleen (Case 5); D, E: Gopher tortoise (*Gopherus polyphemus*), nasal discharge direct smear (Case 6) with spirilliform bacteria consistent with *Helicobacter* sp. in the background, with image E showing abundant extracellular projections presumably consolidated from multiple heterophils; F, G: Gopher tortoise (*Gopherus polyphemus*), swab of oral mucosa; H: Desert kingsnake (*Lampropeltis getula splendida)*, fine needle aspirate of skin lesion; I: Gopher tortoise (*Gopherus polyphemus*), nasal discharge. x100 objective, Wright-Giemsa stain.

An adult male gopher tortoise (*G. polyphemus*) from a zoological facility was presented for nasal discharge and respiratory distress. A whole-body CT scan showed the nasal cavities and bullae were diffusely fluid filled. There were no significant hematological and plasma biochemical abnormalities. Due to lack of response to treatment and clinical decline, the tortoise was euthanized. On gross necropsy, the pharynx and tongue were covered by an extensive, pale tan, soft, pseudomembrane that covered an ulcerated mucosa. There was approximately 12 mL of clear, red, watery fluid in the coelomic cavity. A tissue imprint of the oropharynx revealed marked heterophilic, histiocytic, and lymphoplasmacytic inflammation with mixed bacterial infection and presence of occasional heterophil projections (Figure 3A,B). Cytology of the spleen identified reactive lymphoid hyperplasia as well as a heterophilic infiltrate with few heterophil projections (Figure 3C). Histopathology showed granulocytic infiltration, epithelial hyperplasia and hypertrophy, pigmented macrophage infiltration, and lung congestion. There was fibrinous and necrotic, severe, diffuse splenitis, ulcerative and heterophilic, severe glossitis, pharyngitis, and esophagitis with a fibrinonecrotic membrane, exudative and heterophilic, severe, rhinitis with erosions and necrosis. A choanal swab from this animal was positive for a *Helicobacter* sp. by qPCR (Desiderio et al. 2020). The final diagnosis was severe exudative rhinitis, pseudomembranous glossitis, pharyngitis, and esophagitis, fibrinonecrotic splenitis, necrotizing vasculitis, and colitis/cloacitis.

### 2.6. Case 6

An adult female gopher tortoise (*G. polyphemus*) was presented by a wildlife rehabilitation facility for dull mentation, bilateral nasal discharge, and sunken, closed eyes. On physical examination, the tortoise was in poor body condition with diffuse muscle wasting. Blood analysis was not performed. Cytology of nasal discharge showed moderate heterophilic rhinitis, frequent diplococci, frequent spirilliform bacilli (presumptive *Helicobacter* sp.), mixed bacilli, well-differentiated squamous epithelium, and several heterophils exhibiting whip-like projections with associated extracellular aggregates of presumed heterophil granules (Figure 3D,E). The tortoise was subsequently euthanized. On necropsy, the tortoise was in poor nutritional condition with diffuse muscle wasting and no grossly visible adipose stores. There was concave deformation of the nares bilaterally, and the eyes were sunken with the palpebrae of both eyes fused shut. The liver was diffusely markedly decreased in size. A choanal swab from this animal was positive for a *Helicobacter* sp. by qPCR (Desiderio et al. 2020). Histopathology revealed severe granulocytic and exudative rhinitis with associated spiral-shaped bacteria and mucosa associated lymphoid hyperplasia, lymphocytic and granulocytic tracheitis and bronchitis with mucosal epithelial hyperplasia, lymphocytic and granulocytic interstitial pneumonia with type II pneumocyte hyperplasia and interstitial edema, and a fungal pulmonary granuloma. The final diagnosis was severe nasal helicobacteriosis.

### 2.7. Case 7

An adult male gopher tortoise (*G. polyphemus*) from a zoological facility was presented for nasal discharge and respiratory distress. On physical examination, the patient had severe blepharedema and bilateral nasal discharge. There was moderate leukopenia (4.9 G/L; RI: 10-22 G/L) suggestive of increased peripheral demand, as well as mild hyperproteinemia (49 g/L; RI: 13–46 g/L) and mild hypermagnesemia (5.7 mEq/L; RI: 3.3–4.8 mEq/L), indicative of dehydration (Gibbons et al. 2019). CT scan revealed sand opacity in the caudal gastrointestinal tract and urinary calculi. Despite treatment, the patient continued to decline, was euthanized, and a necropsy performed. Grossly, there was flocculent coelomic effusion, and the lungs were mottled and deep pink in color. Cytology of oral mucosa revealed moderate heterophilic and lesser lymphocytic stomatitis with occasional heterophil projections (Figure 3F,G), presence of frequent mixed bacilli and diplococci, and well-differentiated squamous epithelium. Choanal swab from this animal was positive for a *Helicobacter* sp. by qPCR (Desiderio et al. 2020). On histopathology, there was chronic, perivascular, lymphohistiocytic meningitis with mild hemorrhage and infiltrates of hemosiderophages, granulocytic and lymphohistiocytic interstitial infiltrates, lymphocytic and granulocytic infiltration of trachea, tongue, and esophagus, lymphocytic, plasmacytic, and granulocytic rhinitis, adenitits, and cellulitis, and lympho-histiocytic and granulocytic conjunctivitis and periocular adenitis.

### 2.8. Case 8

An adult male desert kingsnake (*Lampropeltis getula splendida)* was presented for a 3-month history of multifocal skin swellings. On physical examination, the snake had multiple, less than 1 cm cutaneous and subcutaneous swellings located sporadically over the dorsum and lateral body wall. Some of the swellings appeared scabbed. There was no appreciable abnormality of any ventral scales. The exam was otherwise unremarkable. Blood analysis was not performed. Samples of the swellings were obtained by fine needle aspiration. Cytologically, there was marked histiocytic and heterophilic inflammation with mild lymphoplasmacytic component. Many heterophils exhibited whip-like cytoplasmic projections (Figure 3H). There was moderate chronic hemorrhage and presence of pigmented macrophages, and mildly atypical adipose tissue. Histopathology confirmed these lesions to be granulomas with gram-positive bacterial organisms found within the core.

### 2.9. Case 9

An adult male free-ranging gopher tortoise (*G. polyphemus*) was presented after being found lethargic outside of its burrow. On physical examination, it had increased respiratory effort and mucoid nasal discharge. On complete blood count, there was a moderate leukopenia (3.3 G/L; RI: 10–22 G/L), suggestive of increased peripheral demand from inflammation (Gibbons et al. 2019). Chemistry revealed elevated creatine kinase activity (1833 U/L; RI: 32–628 U/L) (Gibbons et al. 2019). CT scan showed periorbital osteolysis, a left single pulmonary nodule, multifocal left unstructured interstitial pulmonary pattern, and pleural and septal thickening. A cytology sample of nasal discharge showed marked heterophilic rhinitis with moderate lympho-plasmacytic and histiocytic components and mixed bacterial infection. Some heterophils exhibited whip-like projections (Figure 3I). A choanal swab was positive for *Helicobacter* sp. by qPCR (Desiderio et al. 2020). A choanal swab was positive for *Mycoplasma* sp. by PCR. Due to clinical decline, the tortoise was euthanized and necropsy was performed. Histopathology showed severe, erosive, ulcerative, granulocytic, histiocytic, and lymphoplasmacytic rhinitis with spiral bacteria and granulocytic, histiocytic, and lymphoplasmacytic interstitial pneumonia.

## 3. Discussion

This descriptive case series is the first to report presumptive HETs in cytology specimens from non-avian reptile patients with various inflammatory conditions as confirmed by necropsy, histopathology, and/or molecular diagnostics; the cytological identification of HETs suggests an active cellular process *in vivo* in response to bacterial and/or viral infection. Further diagnostic testing regarding confirmation of HETs is currently only available in research settings and has thus far only been confirmed in *in vitro* studies (Chuammitri et al. 2009). These techniques (i.e., immunocytochemistry, electron microscopy) were not feasible in clinical cases of this study since sample preparation of fresh samples with advanced techniques and validated antibodies would have been required at the time of sampling, when it was unknown that HETs were present in these cases.

NETs appear to play an important role in host defense from invading pathogens (Yipp et al. 2012; Niedźwiedzka-Rystwej et al. 2019). However, they have also been implicated in several non-infectious disease processes; NETs appear to have pro-inflammatory effects and presumptively perpetuate diseases such as cystic fibrosis, systemic lupus erythematous, and rheumatoid arthritis, and have been associated with certain neoplastic diseases (Farrera and Fadeel 2013; Yu and Su 2013; Sur Chowdhury et al. 2014; Yang et al. 2016; Papayannopoulos 2018; Niedźwiedzka-Rystwej et al. 2019; Twaddell et al. 2019). NETs may impact both pathogen and host; they correlate strongly with disseminated intravascular coagulation and mortality in critically ill humans, providing prognostic information, as patients with increased NET formation required more cardiovascular support, were more likely to have multisystem organ failure, and had higher mortality rates (Abrams et al. 2019).

Systemic inflammation can be challenging to diagnose in reptile patients based on hematological and plasma biochemical analysis and other diagnostic testing. Presumptive HETs were seen in cytology specimens of patients with (n = 3) and without inflammatory (n = 3) leukograms for which hematology data were available; however, the reptilian inflammatory response can be variable and influenced by a variety of intrinsic and extrinsic factors (Stacy et al. 2011). In humans, higher numbers of NETs were found in patients with sepsis compared to non-septic patients, despite there being no significant correlation between NET formation with white blood cell and neutrophil counts (Abrams et al. 2019). Additionally, HETs in this case series were found in cytology samples where no infectious organisms were obviously identified without further diagnostics (e.g., histopathology or culture). This may have been influenced by recent antimicrobial administration, or in cases of viral infections, the poor sensitivity of identifying viral inclusions cytologically. Interestingly, eight out of nine cases had some form of respiratory disease, but the clinical significance of this is unknown at this time.

Both of the pythons with HETs had serpentovirus infections. Of the six gopher tortoises, five of six tested were positive for *Helicobacter* sp., two of two tested positive for *Mycoplasma* sp., and one was positive for both. The caseload from which these were identified included 14 gopher tortoises (n = 6 with HETs) and 12 snakes (n = 3 with HETs). The finding of HETs in association with specific pathogens in these cases is noteworthy. Serpentoviruses are in the order Nidovirales, with other viruses in this order including Coronaviridae and Arteriviridae. NETs have been associated with pathophysiological mechanisms of COVID-19 infection in humans (Zuo et al. 2020). The predominant lesions seen in reptiles with *Helicobacter* are consistent with sepsis, and are otherwise fairly nonspecific (Stacy and Wellehan 2010; Desiderio et al. 2020). *Helicobacter* do not appear to be common in normal *Gopherus* sp. nasal flora; two metagenomic surveys of *Gopherus* sp. did not identify *Helicobacter* sp (Weitzman et al. 2018; García-De la Peña et al. 2019). Further investigations of the interactions of serpentoviruses and *Helicobacter* sp. with pathways of HET induction are indicated.

While further research is needed to fully understand the role and clinical significance of HETs in reptile patients, it is possible that their recognition on cytology samples may be a helpful marker of systemic inflammation, possibly in association with an underlying pathogen resulting in local infection, since these species often lack other objective indicators of inflammation (e.g., leukogram changes). Upon identification of presumptive HETs in cytology specimens from reptile patients, consideration of additional diagnostics to further investigate causes of infection and/or inflammation in reptiles, such as blood culture, tissue cultures, or molecular diagnostics, may be warranted in context of clinical and other pertinent diagnostic findings. Prospective studies using biomarkers of inflammation (e.g., erythrocyte sedimentation rate, haptoglobin, plasma protein electrophoresis) and correlation of HET formation in blood and cytology samples of reptile patients are needed to advance our understanding of clinical relevance and underlying mechanisms of inflammatory responses.

## References

[CIT0001] Abrams ST, Morton B, Alhamdi Y, Alsabani M, Lane S, Welters ID, Wang G, Toh CH. 2019. A novel assay for neutrophil extracellular trap formation independently predicts disseminated intravascular coagulation and mortality in critically ill patients. Am J Respir Crit Care Med. 200(7):869–880. 31162936 10.1164/rccm.201811-2111OC PMC6812439

[CIT0002] Brinkmann V, Reichard U, Goosmann C, Fauler B, Uhlemann Y, Weiss DS, Weinrauch Y, Zychlinsky A. 2004. Neutrophil extracellular traps kill bacteria. Science. 303 (5663):1532–1535. 15001782 10.1126/science.1092385

[CIT0003] Brinkmann V, Zychlinsky A. 2012. Neutrophil extracellular traps: Is immunity the second function of chromatin? J Cell Biol. 198(5):773–783. 22945932 10.1083/jcb.201203170 PMC3432757

[CIT0004] Chuammitri P, Ostojić J, Andreasen CB, Redmond SB, Lamont SJ, Palić D. 2009. Chicken heterophil extracellular traps (HETs): novel defense mechanism of chicken heterophils. Vet Immunol Immunopathol. 129(1-2):126–131. 19178950 10.1016/j.vetimm.2008.12.013

[CIT0005] Desiderio TM, Stacy NI, Ossiboff RJ, Iredale M, Archer LL, Alexander AB, Heard DJ, Crevasse SE, Fredholm DV, Donnelly KA, et al. 2020. Identification of a novel mortality-associated *Helicobacter* species in gopher tortoises (*Gopherus polyphemus*), qPCR test development and validation, and an epidemiologic survey. Proceedings, American Association of Zoo Veterinarians. AAZV Conference 2020. Sept 21–24, 2020.

[CIT0006] Farrera C, Fadeel B. 2013. Macrophage clearance of neutrophil extracellular traps is a silent process. J Immunol. 191(5):2647–2656. 23904163 10.4049/jimmunol.1300436

[CIT0007] García-De la Peña C, Rojas-Domínguez M, Ramírez-Bautista A, Vaca-Paniagua F, Díaz-Velásquez C, Ávila-Rodríguez V, Valenzuela-Núñez LM, Meza-Herrera CA. 2019. Microbiota bacteriana oral de la tortuga del bolsón *Gopherus flavomarginatus* en la Reserva de la Biosfera Mapimí, México. RevMexBiodiv. 90 (2019): e902683.doi: 10.22201/ib.20078706e.2019.90.2683

[CIT0008] Gibbons PM, Whitaker BR, Carpenter JW, McDermott CT, Klaphake E, Sladky KK. 2019. Hematology and biochemistry tables. In: Divers SJ, Stahl SJ, editors. Mader’s reptile and amphibian medicine and surgery. St. Louis (MS): Elsevier; p. 333–350.

[CIT0009] Hoon-Hanks LL, Ossiboff RJ, Bartolini P, Fogelson SB, Perry SM, Stöhr AC, Cross ST, Wellehan JFX, Jacobson ER, Dubovi EJ, et al. 2019. Longitudinal and cross-sectional sampling of serpentovirus (nidovirus) infection in captive snakes reveals high prevalence, persistent infection, and increased mortality in pythons and divergent serpentovirus infection in boas and colubrids. Front Vet Sci. 6(6):338. 31632990 10.3389/fvets.2019.00338 PMC6786048

[CIT0010] Lacerda LC, Lima dos Santos J, Wardini AB, Nascimento da Silva A, Santos AG, Freire HPS, Oliveira dos Anjos D, Romano CC, Mendes EA, Munhoz AD. 2019. *Toxoplasma gondii* induces extracellular traps release in cat neutrophils. Exp Parasitol. 107770. 10.1016/j.exppara.2019.107770. 31586454

[CIT0011] Lawson C, Smith SA, O'Brien M, McMichael M. 2018. Neutrophil extracellular traps in plasma from dogs with Immune-mediated hemolytic anemia. J Vet Intern Med. 32(1):128–134. 29214674 10.1111/jvim.14881 PMC5787156

[CIT0012] Li RHL, Ng G, Tablin F. 2017. Lipopolysaccharide-induced neutrophil extracellular trap formation in canine neutrophils is dependent on histone H3 citrullination by peptidylarginine deiminase. Vet Immunol Immunopathol. 193-194:29–37. 29129225 10.1016/j.vetimm.2017.10.002

[CIT0013] Niedźwiedzka-Rystwej P, Repka W, Tokarz-Deptuła B, Deptuła W. 2019. “In sickness and in health” - how neutrophil extracellular trap (NET) works in infections, selected diseases and pregnancy. J Inflamm (Lond). 16:15. 31297037 10.1186/s12950-019-0222-2 PMC6599315

[CIT0014] Palic D, Ostojic J, Andreasen CB, Roth JA. 2007. Fish cast NETs: neutrophil extracellular traps are released from fish neutrophils. Dev Comp Immunol. 31(8):805–816. 17222907 10.1016/j.dci.2006.11.010

[CIT0015] Papayannopoulos V. 2018. Neutrophil extracellular traps in immunity and disease. Nat Rev Immunol. 18(2):134–147. 28990587 10.1038/nri.2017.105

[CIT0016] Rebordão MR, Carneiro C, Alexandre-Pires G, Brito P, Pereira C, Nunes T, Galvão A, Leitão A, Vilela C, Ferreira-Dias G. 2014. Neutrophil extracellular traps formation by bacteria causing endometritis in the mare. J Reprod Immunol. 106:41–49. 25218891 10.1016/j.jri.2014.08.003

[CIT0017] Rousselet E, Stacy NI, Rotstein DS, Waltzek TB, Griffin MJ, Francis‐Floyd R. 2018. Systemic *Edwardsiella tarda* infection in a Western African lungfish (*Protopterus annectens*) with cytologic observation of heterophil projections. J Fish Dis. 41(9):1453–1458. 29882594 10.1111/jfd.12831

[CIT0018] Stacy BA, Wellehan JF. Jr. 2010. Fatal septicemia caused by Helicobacter infection in a pancake tortoise (Malacochersus tornieri). J Vet Diagn Invest. 22(4):660–662. 20622247 10.1177/104063871002200431

[CIT0019] Stacy NI, Alleman AR, Sayler KA. 2011. Diagnostic hematology of reptiles. Clin Lab Med. 31(1):87–108. 21295724 10.1016/j.cll.2010.10.006

[CIT0020] Stacy NI, Fredholm DV, Rodriguez C, Castro L, Harvey JW. 2017. Whip-like heterophil projections in consecutive blood films from an injured gopher tortoise (*Gopherus polyphemus*) with systemic inflammation. Vet Q. 37(1):162–165. 28460581 10.1080/01652176.2017.1325538

[CIT0021] Sur Chowdhury C, Giaglis S, Walker UA, Buser A, Hahn S, Hasler P. 2014. Enhanced neutrophil extracellular trap generation in rheumatoid arthritis: analysis of underlying signal transduction pathways and potential diagnostic utility. Arthritis Res Ther. 16(3):R122. 24928093 10.1186/ar4579 PMC4229860

[CIT0022] Twaddell SH, Baines KJ, Grainge C, Gibson PG. 2019. The emerging role of neutrophil extracellular traps in respiratory disease. Chest. 156(4):774–782. 31265835 10.1016/j.chest.2019.06.012

[CIT0023] Vargas A, Boivin R, Cano P, Murcia Y, Bazin I, Lavoie J-P. 2017. Neutrophil extracellular traps are downregulated by glucocorticosteroids in lungs in an equine model of asthma. Respir Res. 18(1):207. 29233147 10.1186/s12931-017-0689-4 PMC5727947

[CIT0024] Weitzman CL, Sandmeier FC, Tracy CR. 2018. Host species, pathogens and disease associated with divergent nasal microbial communities in tortoises. R Soc Open Sci. 5(10):181068. 30473851 10.1098/rsos.181068 PMC6227988

[CIT0025] Yang H, Biermann MH, Brauner JM, Liu Y, Zhao Y, Herrmann M. 2016. New Insights into neutrophil extracellular traps: mechanisms of formation and role in inflammation. Front Immunol. 7:302. 27570525 10.3389/fimmu.2016.00302 PMC4981595

[CIT0026] Yipp BG, Petri B, Salina D, Jenne CN, Scott BNV, Zbytnuik LD, Pittman K, Asaduzzaman M, Wu K, Meijndert HC, et al. 2012. Infection-induced NETosis is a dynamic process involving neutrophil multitasking in vivo. Nat Med. 18(9):1386–1393. 22922410 10.1038/nm.2847 PMC4529131

[CIT0027] Yu Y, Su K. 2013. Neutrophil extracellular traps and systemic lupus erythematosus. J Clin Cell Immunol. 04(02):139. 10.4172/2155-9899.1000139 PMC3826916 24244889

[CIT0028] Zhao W, Fogg DK, Kaplan MJ. 2015. A novel image-based quantitative method for the characterization of NETosis. J Immunol Methods. 423:104–110. 26003624 10.1016/j.jim.2015.04.027 PMC4522197

[CIT0029] Zuo Y, Yalavarthi S, Shi H, Gockman K, Zuo M, Madison JA, Blair CN, Weber A, Barnes BJ, Egeblad M, et al. 2020. Neutrophil extracellular traps in COVID-19. JCI Insight. 5(11):e138999. 10.1172/jci.insight.138999 PMC7308057 32329756

